# Responses of Aboriginal and Torres Strait Islander Primary Health-Care Services to Continuous Quality Improvement Initiatives

**DOI:** 10.3389/fpubh.2015.00288

**Published:** 2016-01-21

**Authors:** Sarah Larkins, Cindy E. Woods, Veronica Matthews, Sandra C. Thompson, Gill Schierhout, Maxwell Mitropoulos, Tania Patrao, Annette Panzera, Ross Stewart Bailie

**Affiliations:** ^1^College of Medicine and Dentistry, James Cook University, Townsville, QLD, Australia; ^2^Anton Breinl Research Centre for Health Systems Strengthening, Australian Institute for Tropical Health and Medicine, Townsville, QLD, Australia; ^3^College of Medicine and Dentistry, James Cook University, Cairns, QLD, Australia; ^4^Menzies School of Health Research, Brisbane, QLD, Australia; ^5^Western Australian Centre for Rural Health (WACRH), The University of Western Australia, Crawley, WA, Australia; ^6^School of Medicine, University of Queensland, Brisbane, QLD, Australia

**Keywords:** Aboriginal, Australia, best practice, Indigenous health services, primary health care, quality improvement, quality of care, Torres Strait Islander

## Abstract

**Background:**

Indigenous primary health-care (PHC) services participating in continuous quality improvement (CQI) cycles show varying patterns of performance over time. Understanding this variation is essential to scaling up and sustaining quality improvement initiatives. The aim of this study is to examine trends in quality of care for services participating in the ABCD National Research Partnership and describe patterns of change over time and examine health service characteristics associated with positive and negative trends in quality of care.

**Setting and participants:**

PHC services providing care for Indigenous people in urban, rural, and remote northern Australia that had completed at least three annual audits of service delivery for at least one aspect of care (*n* = 73).

**Methods/design:**

Longitudinal clinical audit data from use of four clinical audit tools (maternal health, child health, preventive health, Type 2 diabetes) between 2005 and 2013 were analyzed. Health center performance was classified into six patterns of change over time: consistent high improvement (positive), sustained high performance (positive), decline (negative), marked variability (negative), consistent low performance (negative), and no specific increase or decrease (neutral). Backwards stepwise multiple logistic regression analyses were used to examine the associations between health service characteristics and positive or negative trends in quality of care.

**Results:**

Trends in quality of care varied widely between health services across the four audit tools. Regression analyses of health service characteristics revealed no consistent statistically significant associations of population size, remoteness, governance model, or accreditation status with positive or negative trends in quality of care.

**Conclusion:**

The variable trends in quality of care as reflected by CQI audit tools do not appear to be related to easily measurable health service characteristics. This points to the need for a deeper or more nuanced understanding of factors that moderate the effect of CQI on health service performance for the purpose of strengthening enablers and overcoming barriers to improvement.

## Introduction

In Australia, there are disparities in health outcomes, with lower life expectancy and high rates of morbidity among Aboriginal and Torres Strait Islander populations and rural and remote populations (1). Primary health care (PHC) is the cornerstone of the Australian health system and provides first contact, continuing ambulatory health-care services. In rural and remote settings, characterized by geographic dispersion and workforce shortages, PHC can be delivered by a range of health-care providers, including resident general practitioners, remote area nurses, Indigenous Health Workers, and fly-in, fly-out services among others.

In this context, improving the quality and consistency of PHC provided to Aboriginal and Torres Strait Islander people is an essential part of the Australian Government’s Close the Gap program (2). The aim of the Closing the Gap policy is to achieve equality in health status and life expectancy between Aboriginal and Torres Strait Islander peoples and non-Indigenous Australians. A range of Indigenous^1^ PHC centers [both Aboriginal Community Controlled Health Services (ACCHS) and government-run health services] provide PHC services for Indigenous people. However, the quality of care provided by such services and the intermediate health outcomes achieved vary significantly between services, as does the response to continuous quality improvement (CQI) activities (3, 4).

Continuous quality improvement aims to facilitate ongoing improvement in the quality of PHC care by using objective information to analyze and improve systems, processes, and outcomes (5, 6). A CQI cycle of “plan, do, study, act” provides a theoretically coherent and practical way for PHC services to organize themselves and engage staff to identify, address, and overcome the barriers to innovation (7). Key features of modern CQI approaches make them well suited to the Indigenous Australian setting (6). The participatory approach and “customer focus” of CQI and the combination of scientific and humanistic values (8–10) accord with the principles and values of Aboriginal and Torres Strait Islander people as expressed in national statements on research and cultural respect (11, 12).

Previous research by Schierhout et al. has illustrated the complexity of the interaction between contexts and mechanisms in mediating response to CQI activities (13), but limited research has systematically investigated the characteristics and contextual factors of a particular service that influence the success (or otherwise) of quality improvement initiatives (2, 3). Yet, understanding this variability in response is a vital next step to facilitate scaling up and sustaining CQI interventions and improvement in the quality of primary care on a broader scale.

Informed by current CQI theory and practice, the Audit and Best Practice for Chronic Disease (ABCD) project demonstrated that a CQI model could be effective in supporting Indigenous PHC services to use evidence-based best practice in chronic illness care, with all 12 participating services achieving significant improvements in clinical systems development, process of diabetes care, and patient outcomes (14).

Following this success, an extension project used action research to explore the potential transferability and sustainability of the model. This extended and improved the audit tools, processes, and resources available to support services and enable development and implementation of additional audit tools beyond diabetes (Maternal Health, Child Health, Rheumatic Heart Disease, and Mental Health) (15). In November 2009, a service support organization was established: One21seventy, which expanded the opportunity for PHC services to engage in CQI beyond research involvement. The name One21seventy reflects the center’s commitment to increasing life expectancy for Aboriginal and Torres Strait Islander people beyond 1 year in infancy, 21 years in youth, and 70 years in the lifespan.

The ABCD/One21seventy CQI cycle uses annual cycles of assessment and feedback using clinical and systems quality improvement tools and a web-based data entry and reporting system to support staff to identify “gaps” in clinical care and health service systems that should be addressed to enhance quality of care. One21seventy provides tools, training, and support for PHC providers to use the CQI audit cycle and a website that facilitates automated analysis and data reports for immediate access by health center staff.

As part of the CQI cycle, a Systems Assessment Tool (SAT) is completed by each participating service, ideally though a group meeting involving clinical staff, for a structured assessment of strengths and weaknesses of the systems in place to support client care. The SAT was developed through modification of the Assessment of Chronic Illness Care scale [based on the Chronic Care Model (16)] (17) and is used by primary health center staff as both a measurement and developmental tool. The SAT enables health service staff to score their health service systems across various domains necessary for effective care and to justify this score. Following the audits and SAT, health services are encouraged to hold a feedback workshop to reflect on and discuss these findings. The health service staff, including non-clinical staff, are then encouraged to collectively set goals and to develop and implement an action plan [goal setting and action plan (GSAP)] over the next 12 months with the aim of enhancing the quality of client care before the next audit cycle.

### The Audit and Best Practice for Chronic Disease National Research Partnership

The ABCD National Research Partnership was established in 2010 to provide ongoing support for services implementing CQI and to continue the program of research on quality improvement in Indigenous PHC. The Partnership works alongside One21seventy to develop the evidence base for quality improvement work. By December 2014, there were 175 PHC services participating in the ABCD NRP (137 government health services, 38 ACCHSs).^2^ One21seventy/ABCD clinical audits are usually completed by clinical staff (nurses, general practitioners, and Indigenous health workers), who have been trained in the use of the audit tools, with support from quality improvement facilitators (18).

#### Response to CQI

Previous research using ABCD/One21seventy audit data has focused on performance over time in diabetes care (18), variation between services in delivery of preventive care (19), improvement in delivery of rheumatic heart disease care (20), barriers and enablers to the implementation of the Plan-Do-Study-Act cycles (21), and a realist analysis of change over time drawing from an evaluation of patterns of change over time (13).

A striking feature of these analyses to date is that some services markedly improved their quality of care as measured by clinical audit through this process, while others had more mixed outcomes showing inconsistent, unchanged or an apparent decline in performance against the best practice standards (4). Analyses suggest that consistent improvement across CQI cycles is not directly related to size of service, remoteness, or type of service; however, at least for diabetes, duration of participation in CQI appears to be important (18).

This paper aims to (i) examine audit performance trends for all services participating in the ABCD National Research Partnership and describe trends in quality of care over time and (ii) examine the association of health service characteristics with positive or negative trends.

## Materials and Methods

### Study Design

This study used an analysis of longitudinal CQI data between 2005 and 2013 to categorize services according to their patterns of change in quality of care over time. Then, backwards stepwise multiple logistic regression analyses were used to examine the associations between patterns of change and health service characteristics.

### Data Collection

CQI data derived from the One21seventy clinical audit tools were available from PHC services participating in the ABCD National Research Partnership between the years 2005 and 2013. Only services that had conducted at least three annual audits were used in this analysis, as this allowed assessment of trends over time. The audit data reflect service delivery as recorded in medical records.

### Data Analysis

#### Performance Scores

Health service performance was calculated for each audit using a summary score of adherence to guideline-derived best practice in service delivery (4, 13). The overall adherence to delivery of scheduled services for each client was calculated by dividing the sum of services delivered by the total number of scheduled services and expressing this as a percentage. For example, if only half the recommended services were recorded as delivered to a client over a particular audit period, overall adherence/performance would be 50%. A mean adherence (for all clients audited) for delivery of Type 2 diabetes care, preventive care, maternal care, and child care in a given health service represented an overall performance score for the health service in a given audit cycle.

Table 1 identifies the clinical indicators for each audit tool used to assess actual practice against best practice standards. Indicators for three of the four audit tools included in this study are identical across states and territories. The number of clinical indicators for the child health audit tool varies between states, ranging from 17 indicators in South Australia to 21 in Queensland and Western Australia, based on different recommended items in child health checks within each jurisdiction.

**Table 1:** **Clinical indicators by audit tools**.

*Type 2 diabetes* 15 clinical indicators	**Physical examination:** weight, waist circumference, body mass index (BMI), blood pressure, visual acuity **Laboratory investigation:** albumin to creatinine ratio (ACR), glomerular filtration rate, blood lipids, Hemoglobin A1c (HbA1c) **Vaccinations:** flu, pneumococcal **Counseling for risk factors:** nutrition, physical activity, tobacco use, alcohol use
*Preventive health* 13 clinical indicators	**Physical examination:** weight, waist circumference, blood pressure, blood glucose level (BGL), oral health check **Laboratory investigation:** urinalysis, sexually transmitted infections (STI) checks – gonorrhea and chlamydia, syphilis serology **Counseling for risk factors:** nutrition, physical activity, tobacco use, and alcohol use **Gender specific checks:** pap smear
*Maternal health* 38 clinical indicators	**Physical examination:** weight (before 13 weeks; 13–26 weeks), body mass index (before 13 weeks), blood pressure (before 13 weeks; 13–26 weeks; post 26 weeks) **Fetal examinations:** fundal height (before 13 weeks; post 26 weeks), fetal heart rate (before 13 weeks; post 26 weeks) **Laboratory investigation:** urinalysis (before 13 weeks; 13–26 weeks; post 26 weeks), blood group, antibodies, full blood examination, rubella, Hepatitis B, syphilis serology, HIV, fetal anomaly test discussed and offered **History of risk factors:** cigarette use (before 13 weeks; 13–26 weeks), alcohol use (before 13 weeks; 13–26 weeks), illicit drug use (before 13 weeks; 13–26 weeks) **Antenatal discussions:** birthing plans, antenatal education, nutrition, breastfeeding, physical activity, oral health, domestic social environment, social family support, financial situation, housing condition, food security
*Child health* 17–21 clinical indicators 17 (SA) 19 (NSW) 20 (NT) 21 (WA, QLD)	**Physical examination:** weight, height, head circumference, hip examination, ear examination, eye examination, testes examination, oral health check (NT, WA, NSW, QLD), gait (NSW, QLD) **Discussion:** child development, breast feeding, nutrition, SIDS prevention, passive smoking risk, domestic environment, family support, housing condition, injury prevention, infection prevention (NT, WA, SA, QLD), physical and mental stimulation (NT, WA, NSW, QLD), financial situation (NT, WA), food security (WA, QLD)

#### Trends in Performance Over Time

**Figure 1:**
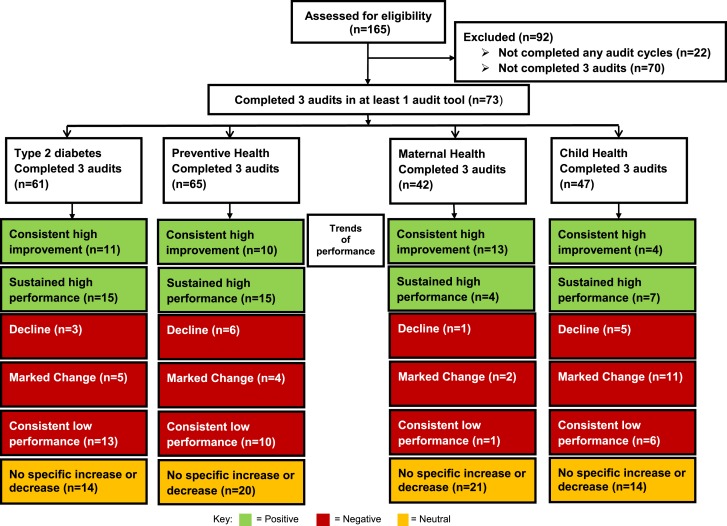
**Health center performance flow diagram**.

**Figure 2:**
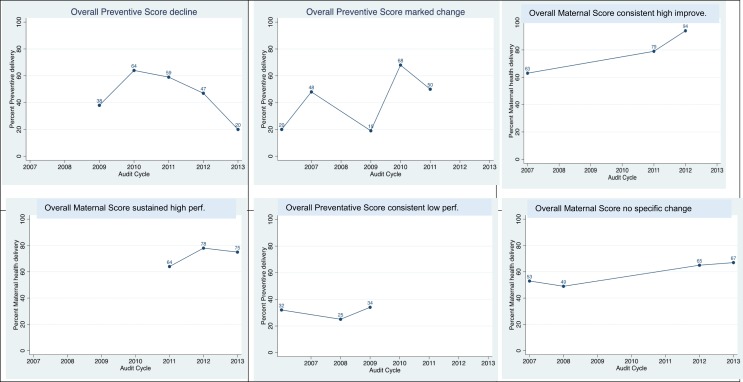
**Examples of six patterns of performance categories**.

Drawing on ABCD CQI clinical audit data for each health service, we constructed a measure of the proportion of guideline-scheduled services delivered for each audit tool in each year of participation. The measure for child health included only services that were specified in the guidelines used in all states/territories where there were participating services. These measures were then represented graphically over the period of participation in each audit tool in panel plots. Based on visual inspection, verified by mathematical analysis (Table 2), six patterns of trends in performance over time were identified: consistent high improvement, sustained high performance, decline, marked variability, consistent low performance, and no specific increase or decrease (Figure 1). Table 2 details the specific quantitative method used to categorize health service trends over time into these six categories for each of the audit tools, and Figure 2 illustrates examples of each of these categories of performance trends over time.

**Table 2:** **Health service performance trends over time**.

Category	Performance trend	Definition	Group for logistic regression analysis
1	Consistent high improvement	Service showed consistent ascending performance scores in an audit from first to last audit and bridged a certain percentage of the gap between the first score and 100% depending on the number of audits it had completed, e.g., if a health service had completed four audits in the maternal audit, then it was required to bridge 30% of the gap (between its first audit performance score and 100%) to be classified as a consistent high improver in maternal health	Positive
2	Sustained high performance	Service demonstrated performance score of its last audits in the top tertile (67th percentile or higher) for a particular audit, e.g., for the Type 2 diabetes audit, the top tertile was a performance score of 73 or higher. For a PHC service that had completed six audits, if the last four performance scores were 73 or higher, it was classified as a sustained high performer	Positive
3	Decline	Service showed consistent descending performance scores from first to last audit and bridged a certain percentage of the gap between the first audit score and 0% depending on the number of audits it had completed, e.g., if a health service had completed four audits, then it was required to bridge 30% of the gap between its first audit performance score and 0% to be classified as a decliner	Negative
4	Marked variability	A PHC service was classified as having a marked variability if the difference between its maximum and minimum performance score was >33% (or a tertile) of its median score, and it showed opposite trends of at least 10% in the preceding and following three audits	Negative
5	Consistent low performance	Services that consistently performed below the baseline median performance of the audit tool	Negative
6	No clear increase or decrease over time (no specific trend)	A PHC service was classified as having no specific increase or decrease over time if its performance score was less than the top tertile in the first audit and not more than a 33% difference (or a tertile) between maximum and minimum, or if the PHC service does not fall under any of the above categories	Neutral

For the purpose of analysis of performance over time and health service characteristics, the categories of trends over time were grouped as positive (consistent high improvement/sustained high performance), neutral (no specific increase or decrease), and negative (decline/consistent low performer/marked variability) (2).

#### Explanatory Variables

Backwards stepwise multiple regression analyses were conducted to identify whether any significant associations exist between performance category of health services and health service characteristics. Health service performance trends (positive or negative) were used as the outcome variable. The independent variables included in the model were governance (government- or community-controlled health center); location [rural, remote, urban based on the Australian Standard Geographical Classification (ASGC-RA) system]; population size (≤500, 501–999, ≥1000); accreditation status during the audit period as reported in the health center and community survey (never accredited, accredited for some of the time, and accredited for all of the time); CQI commencement year; and continuous variables representing the percentage of SAT and Goal Setting and Action Plans (GSAP) completed.

Results are expressed as odds ratios (OR) and 95% confidence intervals (CI). All variables were included in a model and then backwards stepwise multiple logistic regression was used to eliminate some variables and result in a final model. Mann–Whitney *U* tests were used to analyze associations between positive performance trends over time and the number of audits performed. Alpha of <0.05 was considered statistically significant. Statistical analysis was conducted using STATA software, V.13 (StataCorp, College Station, TX, USA).

#### Ethical Considerations

Ethics approval was obtained from research ethics committees in each jurisdiction [Human Research Ethics Committee of the Northern Territory Department of Health and Menzies School of Health Research (HRECEC00153 – HREC 09/97); Central Australian Human Research Ethics Committee (HREC-12-53); New South Wales Greater Western Area Health Service Human Research Committee (HREC/11/GWAHS/23); Queensland Human Research Ethics Committee Darling Downs Health Services District (HREC/11/QTDD/47); South Australian Aboriginal Health Research Ethics Committee (04-10-319); Curtin University Human Research Ethics Committee (HR140/2008); Western Australian Country Health Services Research Ethics Committee (2011/27); Western Australia Aboriginal Health Information and Ethics Committee (111-8/05); University of Western Australia Human Research Ethics Committee (RA/4/1/5051); James Cook University Human Research Ethics Committee (H5541)] to conduct this project.

## Results

Out of the 165 services participating in the ABCD National Research Partnership, 73 (44%) had completed three audits (baseline, years 1 and 2) in at least one audit tool (61 for Type 2 diabetes audit, 65 for preventive audit, 42 for maternal audit, and 47 for child audit; Figure 1).

Forty of the 73 services are located in Queensland, 25 in the Northern Territory, 4 in New South Wales, 3 in Western Australia, and 1 in South Australia; 62 services are in remote locations, with the remainder in urban (*n* = 1) or inner or outer regional (*n* = 10) locations. Eighteen of the eligible health services are ACCHS, and the remainder are state- or territory-operated.

### Classification of Trends in Performance Over Time

Health services commenced participation in CQI at differing times and completed varying numbers of audit cycles. Table 3 shows the number of services using each audit tool by year. The most commonly used audit tools are those for Type 2 diabetes and preventive health (Figure 1).

**Table 3:** **Number of services using each audit tool by year**.

	Number of audits by year			
Year of audit	T2DM	Child health	Maternal health	Preventive health
2005	7	–	–	8
2006	21	–	–	21
2007	21	11	11	23
2008	28	23	17	29
2009	37	28	21	39
2010	32	33	28	36
2011	50	45	35	66
2012	33	43	39	41
2013	22	13	23	29

There was a wide variation in baseline level of performance and divergent trends in performance achieved by health services across the four audit tools. Thirty health services were categorized as consistent high improvers in at least one audit tool (not shown).

Considering performance by audit tool, 43% (*n* = 26) of 61 health services showed consistent high improvement or sustained high performance (i.e., a positive trend) in delivery of diabetes care, 38% (*n* = 25/65) in preventive health, 40% (*n* = 17/42) in maternal health, and 23% (*n* = 11/47) in child health (Figure 1).

No significant associations were found between a positive trend (when compared with negative or neutral trends over time) and number of audits performed for Type 2 diabetes (*p* = 0.59), child health (*p* = 0.60), maternal health (*p* = 0.52), or preventive health (*p* = 0.10).

When services with a positive trend in performance were compared with those with neutral or negative trends, there were no consistent associations across each audit area between accreditation, governance, remoteness, population size, CQI commencement year, percent SAT completed or percent GSAP completed, and health service performance.

Percent of GSAP undertaken over the period of the audits (OR: 0.98; 95%CI 0.96–0.99) is associated with a negative trend in performance in Type 2 diabetes (Table 4). Percent of SAT undertaken over the period of the audits (OR: 0.97; 95%CI 0.95–0.99) is associated with a negative trend in performance in child health (Table 4). None of the health service characteristics are significantly associated with trends in performance in maternal or preventive health.

**Table 4:** **Analyses of health service factors and trends in performance over time**.

CQI audit tool	Health service characteristic	Unadjusted OR	95% CI	*p*-Values
T2DM	% GSAP completed	0.98	0.96–0.99	0.01
Child health	% SAT completed	0.97	0.95–0.99	0.01

## Discussion

This study has demonstrated the feasibility and utility of classifying and describing trends in quality of care over time (defined as the proportion of guideline-derived care delivered) provided by Indigenous PHC services engaged in CQI activity. This allows an analysis of health service characteristics associated with each trend in performance, across each audit tool. To the best of our knowledge, this is the first time trends in performance associated with CQI activity have been empirically developed from large-scale CQI PHC data and used as an outcome measure for health service performance over time. The quantitative classification method used in this study may be of interest to leaders of other CQI programs, most likely as a means of selecting services with different performance patterns for further qualitative exploration.

There is no clear or consistent evidence that any of the health service characteristics that were included in this analysis showed an association with patterns of change in clinical performance. The significant associations that were found for some health service characteristics are counterintuitive, and these associations are not consistently demonstrated in the analysis of data using different audit tools, so clearly further exploration is needed.

The analyses suggest that trends in performance are subject to influences that are more difficult to quantify. There is also a need to improve measurement of some of the influences that may be quantifiable for the purpose of this type of research – for example, accreditation and quality of the CQI process (including rigor in use of SATs, quality of goal setting, and action planning processes).

### Strengths and Limitations of the Study

The strength of this study is the access to a large, geographically distributed CQI dataset involving longitudinal data from 165 services from which patterns of performance can be analyzed and a strong network of Indigenous PHC services, peak bodies, and academic partners across jurisdictions. There are also some important limitations to this analysis which mean that we cannot conclusively say that the factors that we have examined do not have an influence on trends in performance, at least in some contexts. Key limitations include that (a) the sample size in terms of numbers of health services within each audit tool dataset is relatively small (65 health centers within the largest dataset) and therefore numbers within each trend in performance category (positive, negative) are also small; (b) the numbers of services using each tool by year, for the purpose of comparing individuals against the median score for all PHC services, was also relatively small; and (c) some of the health service system measures/indicators (such as GSAP completion) had missing data and may need further development as quantitative measures.

Eligible health services were not randomly selected but were participants in the ABCD NRP. Although this reflects either a commitment to CQI at health service and/or jurisdictional level, the commitment of individual services to the rigor of the protocols varied. Health services commenced participation in the ABCD NRP at different times over a period of years. Thus, duration of participation was longer for some health services than others and the number of audit cycles in each audit tool varied between services, with some services having substantial gaps between audits. The study relied on data retrieved from paper-based and electronic clinical medical records, which may underestimate actual service delivery due to lack of documentation in clinical records.

Although health services are provided with guidelines to achieve an adequate sample size for each audit tool, sampling approaches by individual health services may be affected by available time and resources and may not be representative of the eligible population in each community. The study does not include patient-level characteristics, which have some influence on the level of health service performance in each tool (18).

The finding that there was no consistently significant association between CQI performance trend category and health service level characteristics may reflect the relatively small numbers of PHC services in some groups. Despite being the largest research-oriented database of CQI data, the number of health services within each pattern of change was relatively small limiting statistical power.

### Discussion of the Findings in Relation to Other Relevant Research

The comparability of these findings with similar studies is limited by this being the first study to use trends in service delivery at the health service level over time as an outcome variable rather than adherence to delivery of scheduled services (4, 19). Nonetheless, previous research has found delivery of care is better in remotely located health services (18, 19), health services with smaller service populations, community-controlled services (19), longer participation in the CQI program, and regularity of client attendance (18).

Our finding that accreditation of health services is not associated with a positive trend in quality of care, is consistent with previous research (4, 13, 20, 21) and indicates that improvement in quality of care at the health service level may be related to within service factors (13, 20).

Primary health-care services are both complex adaptive systems themselves and operating within larger complex adaptive health systems (22). PHC services are multidimensional organizations, with a large number of factors potentially interacting in their influence on capacity to respond to CQI, and many of these factors are not easily measured (4, 15). Quality of care provided by individual health services and the degree of response to CQI activities varies widely due not only to within service factors but also community and individual factors, including health-seeking behaviors (4).

The qualitative realist study by Schierhout et al. identified several processes impacting on improved service delivery outcomes that are not easily measurable (13). These factors were (1) collective or shared valuing of clinical data for performance improvement; (2) collective efficacy – a shared belief of achieving improvement through CQI audit processes; and (3) organizational orientation toward population health. These factors are also affected by a range of contextual factors that can act as facilitators or constraints to improved service delivery.

### Implications of Findings for Practice, Policy, and Research

Understanding variations in quality of care and response to CQI initiatives has been an ongoing challenge for health service researchers (23). The variation in delivery of guideline-scheduled services, and particularly health services showing a negative trend in association with CQI activities, highlights important areas for action in these health services and may reflect a lack of commitment to CQI, a lack of leadership and teamwork within the services, or a variety of other possible influences on performance. However, it is critical that the measures listed here not simply be used as part of a performance and outcomes framework by funders without further exploration. The well-known burden of disease in the Indigenous population and the low levels of adherence to clinical guidelines in some health services indicate that important opportunities for delivery of preventive services, early detection and treatment, chronic disease care, and maternal and child health care are being missed.

To achieve population health impact, the next step is to increase understanding of how to lift the standard of care on a broad scale (24). Detailed qualitative studies of services demonstrating positive or negative trends in performance in association with CQI initiatives may help us understand how effective CQI operates within and between services. Further fine grained understanding of system factors and patient-level factors that promote consistent high improvement in quality of care will inform clinicians, health managers, and policy makers to develop strategies to improve quality of care in Indigenous health services.

## Conclusion

Identifying and categorizing trends in performance over time in Indigenous health service performance in association with CQI provides essential information to the health services themselves, to governance and to policy makers in terms of the variable performance of mainly remote Indigenous health services and the number of services that may require additional support to improve the quality of care provided to their clients. Improving health care in Indigenous communities is vital to reduce Indigenous health disparity and contribute toward “Closing the Gap” in terms of health outcomes and life expectancy.

The variable trends in quality of care as reflected by CQI audit tools do not appear to be related to easily measurable health service characteristics. Possibly due to limitations in existing data, analysis of health service characteristics based on trends in performance did not yield any consistent results. As health services continue to engage in the CQI audit process and more data become available this may become a useful method to examine which, if any, health service characteristics are associated with trends in health service performance. Nonetheless, this process was valuable in terms of the development of a quantitative method to identify health services which have showed positive or negative trends in quality of care delivered or little change over time in each of the audit tools.

The lack of consistent associations between service level characteristics and positive and negative trends in association with CQI may be an indication of the complexity of the relationship between service level, network, community, and patient factors in terms of supporting quality improvement. Detailed qualitative case studies with high-improving services are now in progress to provide rich, contextual exploration of how these factors operate and interact in facilitating or limiting quality improvement.

## Author Contributions

The study was conceived and designed by RB, SL, GS, and ST. SL was the principal investigator for the study and the main author of this paper and guarantor. VM, TP, MM, and CW contributed to the data collection and analysis, with advice and guidance from RB, SL, and GS. SL, TP, AP, VM, MM, and CW contributed to the manuscript writing. SL, RB, and ST assisted with the interpretation of the findings and manuscript editing. ST advised on the clinical aspects of the findings. All authors contributed to the synthesis of the overall study findings reported here, and critically revised the manuscript for important intellectual content. All authors have seen and approved the final paper. All authors agree to be accountable for all aspects of the work in ensuring that questions related to the accuracy or integrity of any part of the work are appropriately investigated and resolved.

## Conflict of Interest Statement

The authors declare that the research was conducted in the absence of any commercial or financial relationships that could be construed as a potential conflict of interest.

## Footnotes

^1^When the term Indigenous is used, it refers respectfully to Aboriginal peoples and Torres Strait Islanders.

^2^Aboriginal Community Controlled Health Services are federally funded holistic PHC services governed by an Indigenous community-elected Board of Directors.
